# Effect of Turmeric Concentrations on the Rate of Growth of Oral Bacteria—An In-Vitro Study

**DOI:** 10.3390/dj9030026

**Published:** 2021-03-01

**Authors:** Yun Xuan Yang, Vicky Wu, Hadi Malak, Aliya Peer Ahamed, Aaron Lo, Yannis Abraham, Catherine Miller

**Affiliations:** 1 College of Medicine and Dentistry, James Cook University, Cairns, QLD 4870, Australia; Vicky.Wu@my.jcu.edu.au (V.W.); Hadi.Malak@my.jcu.edu.au (H.M.); Aliya.Peer@my.jcu.edu.au (A.P.A.); Aaron.lo@my.jcu.edu.au (A.L.); 2 College of Public Health, Medical and Veterinary Sciences, James Cook University, Cairns, QLD 4870, Australia; kate.miller1@jcu.edu.au

**Keywords:** turmeric, growth, oral bacteria, in-vitro

## Abstract

Background and Aim: The aim of this study was to evaluate the effect of varying concentrations of a turmeric solution on the growth rates of oral bacteria sampled from dental students. Methods: Bacterial cultures were grown overnight in aerobic conditions from plaque samples obtained from five test subjects. With the exception of the control, samples were exposed to different treatments; including chlorhexidine gluconate 2 mg/mL, prepared turmeric solution (TS) mouthwash: TS 0.25 mL (7.375 mg/mL), TS 0.5 mL (14.75 mg/mL), and TS 1 mL (29.50 mg/mL). Growth rate of the bacterial cultures were assessed by monitoring changes in optical density readings at 600 nm at hourly intervals for a six-hour period. The data were plotted and the exponential trend was used to calculate individual rates of growth. Data was analyzed using a one-way ANOVA with the significance confirmed using the Tukey-HSD test. Results: Growth observed in the bacteria exposed to the turmeric solution, was significantly greater (*p* < 0.05) when compared with the bacteria exposed to the medium alone. There was a significant difference found between the bacterial growth rate of the 1 mL turmeric solution against the growth rate of the bacteria in the 0.25 and 0.5 mL turmeric solutions. Conclusion: Comparison of growth rates of oral bacteria suggested that turmeric solutions of concentrations between 7.357 and 29.5 mg/mL (0.25–1 mL) were unlikely to exhibit bacteriostatic or bactericidal properties, and, conversely, increased bacterial growth. Considering this result, it is unlikely that turmeric mouthwash made from store-bought turmeric would have any antibacterial effects against oral bacteria, and may even promote bacterial growth.

## 1. Introduction

Periodontal diseases are one of the most common dental conditions, affecting approximately 20–50% of the global population, making it a worldwide public health concern [1]. The aetiology of this condition stems from the undisturbed accumulation of dental plaque—a complex biofilm of bacteria from the oral cavity and their by-products—aggregating on the tooth surface [2]. Therefore, plaque-induced gingivitis, the most common form of periodontal disease, can be treated successfully by the removal of plaque, and thorough and frequent oral hygiene practices [3]. Plaque control can be achieved through mechanical and chemical means. Chemical agents are used as an adjunct to oral hygiene practices, and various synthetic chemical agents have been investigated with respect to their antimicrobial activity [4]. Currently, chlorhexidine is widely considered to be the benchmark for plaque control. However, its use is limited to short periods only, due to the associated side effects, including oral mucosal erosion, dysgeusia, and superficial staining of the tongue, tooth surfaces and restorations. Other mouthwashes available on the market today also have a number of side effects and can often be expensive [1,5].

High cost and undesirable side effects associated with commercial medicines, has led to the increasing demand for Complementary and Alternative Medicines (CAM) that are safer, effective, and economical [6]. CAMs represent a group of diverse medical and health care systems, practices, and products that are not considered to be part of evidence-based conventional medicine. The popularity of CAMs stems from a general belief that these medicines are safe since they are derived from natural resources [7]. Common examples include herbal remedies, homeopathic medicines, and essential oils [6]. CAMs remain largely understudied, as the safety, effectiveness, and potential benefits beyond clinical trials are still unclear despite being used for many centuries [7,8]. The dental profession is one that is rooted in evidence-based diagnostic and treatment approaches, and due to the lack of sound evidence supporting CAMs, dental professionals are currently reluctant to recommend natural chemical agents as an adjunct to conventional therapy in clinical practice [8].

Home-made mouth rinses are a natural, easily-accessible, and low-cost alternative to commercial chemical agents, and have been reported to aid in the removal of dental plaque [9]. Cruz and Picazzo [10]. reported that primary dental service is very limited in rural regions of Mexico, so people turn to CAM to treat common oral diseases such dental caries and periodontal disease. One of the more popular options of these alternative solutions, is a turmeric mouthwash preparation. *Curcuma longa* L. is a rhizomatous herb known as turmeric, belongs to the family of Zingiberaceae. Turmeric has been shown to exhibit antimicrobial, anti-inflammatory, and immune-stimulant properties, which reduces bacterial load and subsequently reduces rates of dental caries and gingival inflammation [11,12]. Chaudhari et al [13] found a reduction in the gingival index and plaque index of participants who used turmeric solution, thus concluding turmeric solution is effective in inhibiting dental caries and periodontal disease.

As the link between oral diseases and the activities of microbial species that form part of the microbiota of the oral cavity are well-established, the efficacy of this CAM is plausible [11,12]. Although sufficient literature exists on the antimicrobial and anti-inflammatory properties of curcumin—turmeric’s active ingredient—its antimicrobial effect exerted at a cellular level on oral bacteria cultured in aerobic conditions is not extensively reported. Furthermore, there is no definitive minimum inhibitory concentration for the bacteriostatic effect of store-bought turmeric currently reported. [11,12]. 

Our research project aims to determine the effectiveness of turmeric solutions on the rate of in-vitro bacterial growth. We hypothesize that with increasing turmeric concentration, there will be a decrease in bacterial growth. Our results showed that, contrary to our hypothesis, the tumeric solution stimulated rather than inhibited bacterial growth in vitro. As a result, our research has the potential to expand on existing knowledge within current dental literature regarding turmeric in its various forms and as a CAM. In helping to create a strong evidence-based foundation for the safe and effective use of this natural therapy, dentists will be able to make informed suggestions for patients to use turmeric mouth rinses as a reliable adjunct to conventional treatment as needed.

## 2. Methodology

This is a case control study approved by Human Research Ethics Committee (HREC) at James Cook University (H7803). Biosafety requirements, set by the James Cook University Institutional Biosafety Committee, were met by all members of the investigative team.

Plaque Collection: A total of five plaque samples were collected from volunteers who fit the inclusion criteria (Table 1).

For the research project. To obtain the sample, participants dislodged plaque from the surface of their teeth using Erskine^TM^ sterile pikster swabs. The plaque samples were then dispersed into 1.5 mL microfuge tubes containing 1 mL phosphate buffered saline (PBS) by swirling the pikster until the plaque particles were visibly dispersed in the solutions. The 5 mL test tubes were randomly numbered from one to five. Then, 1 mL of each of the plaque samples were transferred into 15 mL test tubes containing 4.5 mL of Luria-Bertani (LB) broth for overnight culture under aerobic conditions. To produce an aerobic environment, the test tubes were loosely capped and shaken overnight at 37 °C.

Turmeric (Specimen Code: 36610E) Solution Preparation: The most common preparation found on various online CAM blogs for home-made turmeric mouth rinses was 1 teaspoon of turmeric powder in 1 cup of warm water [13,14]. To replicate these conditions, 4 g of turmeric powder was mixed into 250 mL of MilliQ^®^ water to make the initial turmeric stock mixture. The mixture was shaken to ensure even distribution of the turmeric, and a sample of this mixture was filtered through Whatman Filter Paper^®^ #1 into a second container (C1). A total of 52 mL of turmeric mixture was collected in C1. Once the mixture had filtered through, the filter paper was removed and dried overnight. After subtracting the initial weight of the filter paper, the total weight of the filtrate was found to be 131 mg. The solution in C1 was allowed to sit overnight to settle any remaining undissolved turmeric. This was then pipetted out and filtered again, using the same methods as before. Another 7.8 mg of undissolved filtrate was recorded. The turmeric solution was boiled for five minutes, centrifuged, and then allowed to rest for a day. A fully saturated turmeric solution (TS) was assumed, as there was no turmeric sedimentation at the base of the container. 

The second round of filtration resulted in a decrease in total volume of the turmeric stock solution from 52 to 47 mL, with a total of 138.8 mg of filtrate removed from the original solution. To calculate the concentration of TS, it was assumed that the shaking of the initial mixture resulted in an evenly distributed amount of turmeric in the mixture before it was poured through the filter paper. Thus, the original aliquot of TS contained 832.11 mg of turmeric powder in 52 mL. After accounting for the removed turmeric filtrate and the final volume of TS, the final concentration was determined to be 14.75 mg/mL.

**Sample Preparation:** Overnight cultures were diluted 1:10 with fresh LB broth and the treatments added as listed below:

**Group 1**: The test tubes in this group contained 4.5 mL of LB broth and 0.5 mL of the overnight culture as an untreated control.

**Group 2**: The test tubes in this group contained 0.5 mL of undiluted chlorhexidine gluconate 2 mg/mL (Savacol™, Colgate-Palmolive, Australia), 0.5 mL of the overnight culture and 4 mL of LB broth.

**Group 3**: The test tubes in this group contained 0.5 mL of turmeric solution (1×), 0.5 mL of overnight culture, and 4 mL of LB broth.

**Group 4**: The test tubes in this group contained 0.25 mL of turmeric solution (0.5×), 0.5 mL of overnight culture and 4.25 mL of LB broth.

**Group 5**: The test tubes in this group contained 1 mL of turmeric solution (2×), 0.5 mL of overnight culture and 3.5 mL of LB broth.

Another set of solutions were made as described above but without bacteria to be used as blanks when measuring absorbance of the cultures (Table 2).

The test tubes containing the plaque and treatments were returned to 37 °C and incubated (with shaking) for six hours until a plateau in the bacterial culture growth was noted. At time 0, and every 60 min afterwards, 0.1 mL was removed in duplicate after agitation of the test tube. They were placed in the wells of a 96 well plate and the absorbances were read at optical density (OD) 600 nm. Due to the inherent differences in absorbance values of the different treatments, the initial OD_600_ of each of the individual treatments in LB (without added plaque) were subtracted from the total OD_600_ value to accurately visualize the effects of the treatments on the growth of the plaque itself.

The absorbance readings for all the samples were charted using Microsoft Excel. The duplicate samples were used to calculate the average absorbance values for each plaque sample in each treatment, at every hour and for each subject. The OD_600_ readings for the containers with only the treatment in LB were subtracted from the corresponding treatment averages as discussed above. These values were then averaged across all subjects for every treatment to determine the average OD_600_ reading at every hour for each treatment.

**Figure 1:**
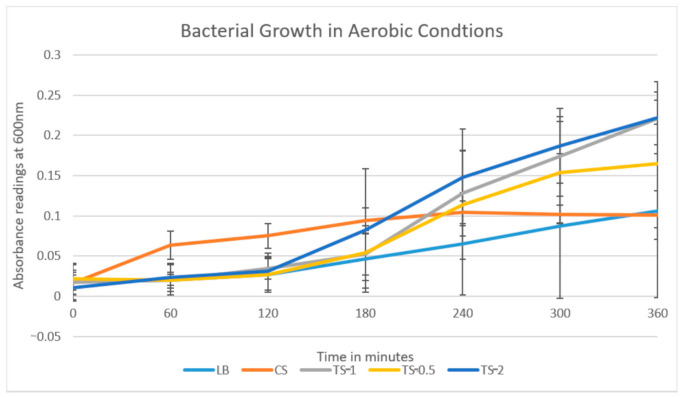
Growth curve of oral bacteria exposed to varying concentrations of turmeric.

Bacterial growth readings were taken and charted until the 360 min mark when bacterial growth was observed to reach a plateau. The experimental data comparing the growth of bacterial samples were plotted and presented in growth curves comparing the ΔOD_600_ of the plaque samples in different treatments over time in minutes (Figure 1).

Bacterial growth occurs in four phases. The initial lag phase consists of limited or slow growth as bacteria acclimate to their new environment. The second logarithmic or exponential phase is where bacteria are most rapidly reproducing. As bacteria most commonly reproduce asexually through binary fission, an exponential curve can be approximated and is characterized graphically by the number of doublings, although less than ideal conditions will result in slower growth. The third saturation or stationary phase is characterized by an equilibrium between the number of dividing and dying cells as nutrients are depleted, and waste products accumulated. The final death phase is the point where bacteria begin to die as toxic, catabolic products accumulate. Dead cells release nutrients into the environment at the end of this phase which surviving bacteria may use for their survival leading to a state of long-term stationary phase lasting from several weeks to months [15].

By analyzing the experimental data plotted over a six hours growth period, the exponential growth phase for the bacteria was determined to start at 120 min (where the lag phase was observed to end) until 360 min. The exponential growth rate (EGR) of the bacteria for each treatment was calculated using the formula: N_T_ = N_0_ (1 + r)^T^ where T = time; N_T_ = the amount of bacteria (absorbance) at time T; N_0_ = the amount of bacteria (absorbance) at the beginning of the exponential phase (T = 0); and r = exponential growth rate in the determined hours of exponential growth. 

The experiment was completed twice, and two sets of EGR for the bacteria in each treatment were obtained, represented as ΔOD_600_/60 min. 

## 3. Statistical Method

In order to analyze the statistical significance of each of the different interventions against the control group (LB), a one-way ANOVA test was performed with a post-hoc analysis using the Tukey–HSD (Honest significant difference) test with a statistical significance of *p* < 0.05. The test was performed on IBM SPSS Statistics 25, and utilized the calculated EGRs from both rounds of testing.

## 4. Results

The results plotted from the first round of testing (Figure 1) and the repeat showed similar trends, with the greatest absorption, and thus bacterial growth, from TS-2, followed by TS-1, TS-0.5, LB, and CS.

Bacterial cultures were set up for each participant as described in the Materials and Methods. The cultures were incubated at 37 °C for 360 min with duplicate aliquots of 0.1 mL removed every 60 min. OD_600_ was determined for each culture for each participant. The results are presented as mean ± SE of all participants for each treatment.

The growth rate of the oral bacteria in the growth medium alone was similar in both rounds of the experiment with no significant growth (Table 3). In both experiments, the turmeric solutions were found to have the highest growth rates, with the rate increasing as the concentrations of turmeric within the solutions increased. TS-0.5 (7.375 mg/mL) displayed a lag phase for the first two hours of the experiment, where it then reached the exponential growth phase for the next three hours, reaching the stationary phase for the last one hour of the experiment beginning at hour five. TS-1 (14.75 mg/mL) similarly displayed two hours lag phase, followed by an exponential phase which continued for the remainder of the experiment. TS-2 (29.50 mg/mL) displayed two hours lag phase followed by an exponential growth for the remainder of the experiment. The lowest growth rate was consistently found to be the bacteria within the CS, with noticeably reduced exponential growth rates compared to the growth medium alone and the turmeric solutions.

**Figure 2:**
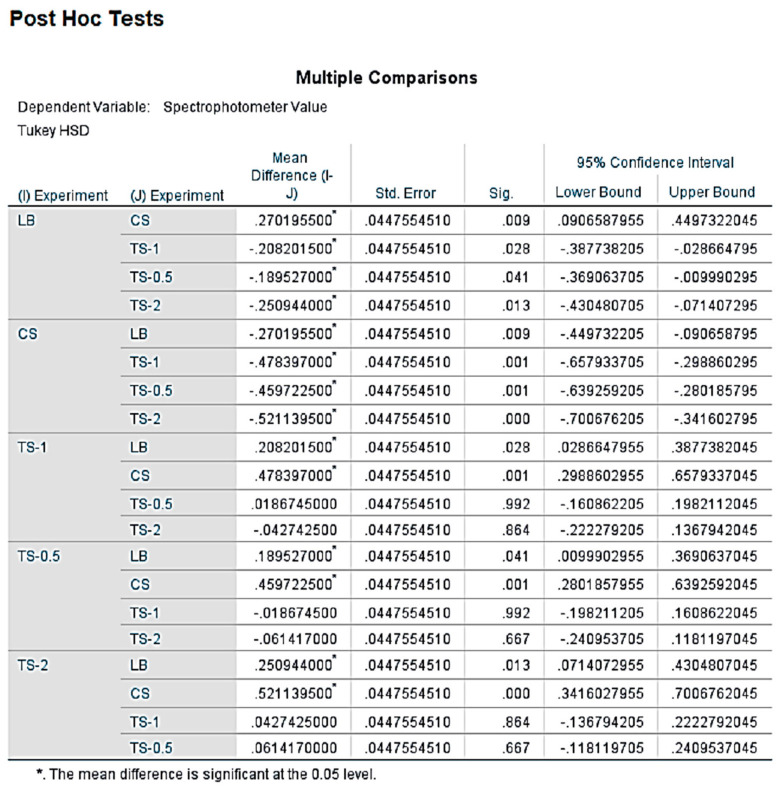
SPSS results using the one-way ANOVA and Tukey Post-Hoc tests revealed that exponential growth rate of samples treated with CS (*p* = 0.006), TS-1 (*p* = 0.005), TS-0.5 (0.018), and TS-2 (0.000) were significant in comparison to the sample exposed to only LB. There was a significant difference (*p* < 0.05) in the exponential growth rates of TS-2 against TS-1 and TS-0.5. However, there was no significance between the EGR of TS-1 and TS-0.5.

Statistical Analysis: The Shapiro–Wilk test and Q-Q plot indicated a normal distribution of our data. As stated above, this has allowed us to perform statistical analysis using a one-way ANOVA test and Tukey Post-hoc tests (Figure 2).

## 5. Discussion

This study monitored bacterial growth in plaque samples placed in Luria Bertani growth medium and exposed to varying concentrations of turmeric solution, chlorhexidine gluconate 2 mg/mL and the growth medium alone, used as a control, over a six-hour time frame.

The overall findings of the study revealed that the addition of turmeric was associated with increasing bacterial growth, with increasing turmeric solution concentration corresponding with an increase in bacterial growth. Furthermore, the growth rates of the bacteria in the turmeric solutions are significantly and consistently higher than our control group solution (bacteria exposed to only LB broth). These results contradict the existing research discussing the anti-inflammatory and anti-bacterial effects of turmeric on the oral microbiota [3]. However, as concluded from our literature review, current research predominantly investigates only the efficacy of Curcumin (diferuloylmethane)—one of the active constituents of turmeric—resulting in a gap in the literature [3,16]. Therefore, in the available studies, the ‘turmeric solution’ was in fact a solution comprised of curcumin extract prepared through pharmaceutical means or through a complex process of obtaining fresh turmeric and treating with ethanol to obtain pure curcumin extract. However, our study was designed to incorporate the Do it yourself (DIY) and home remedy methods that CAM users can readily prepare at home. Store-bought turmeric that is readily available to consumers consists of approximately 60% carbohydrates, 6% water, 6% protein, 5% fat, 3% dietary minerals, 3% essential oils, 2% dietary fiber, and only 1% curcumin [17]. This is in stark contrast to the studies which use 3–5% curcumin from 100 g of turmeric to achieve a minimum inhibitory concentration of the bacteria [3,12,17]. Therefore, to achieve a concentration of active ingredient that is bacteriostatic or bactericidal, we would require a significantly higher amount of store-bought turmeric in solution than what is normally consumed in conventional holistic mouthwashes. Hence, this is a potential explanation for the lack of bacterial inhibition by the turmeric in our experiment. As existing studies use only pure curcumin extract, they do not take into account the additives present in readily accessible turmeric powder and thus do not reflect the practices that the majority of CAMS users employ.

The causative relationship between the increasing turmeric concentrations and the growing bacteria can be a result of the presence of carbohydrates, sugar, and dietary minerals which promote overgrowth of cariogenic bacteria in the dental plaque [18]. In-vitro studies largely conclude that the presence of sucrose and other carbohydrates act as nutrients for the bacteria and provide the required energy for proliferation [18]. Although it cannot be confirmed, we can surmise that these strains were present in our plaque biofilm, as they are most commonly found bacteria. This results in a significant increase in streptococci bacteria when exposed to carbohydrates and water which is also reflected by our results.

Chlorhexidine gluconate 2 mg/mL was used to confirm the validity of the experimental design. Chlorhexidine is often considered the “gold standard” for plaque control mouth-rinses and is known to be a potent broad-spectrum biocide with strong bacteriostatic and bactericidal effects [18]. Although a bacteriostatic phase was eventually achieved, it is important to note that initially during the lag phase, the bacterial samples treated with CS continued to grow. It was also observed however, that the LB sample failed to show a steady increase in growth as was initially expected. Retrospectively, in the absence of the nutrients provided by the store-bought turmeric, the standard method of placing plaque samples in LB growth medium may not create the environment required for the bacteria present in plaque to grow exponentially. Although this method may be effective in growing saliva samples, the specific and complex structural configuration of plaque represents a true biofilm consisting of a variety of microorganisms involved in a wide range of physical, metabolic, and molecular interactions. The cooperative nature of the microbial community allows a greater range for growth. Taking into account in-vitro studies of biofilm structure and function, further repetitions of our experiment can use model systems for which the environmental variables of plaque can be rigorously controlled. For instance, an in-vitro dental plaque model where plaque containing different bacterial strains are placed in a saliva-based medium on hydroxyapatite discs coated with a salivary pellicle [19]. Nevertheless, we do see a steady increase in the growth of bacteria in the growth medium, but due to our limitations, it is at a slower rate than expected.

Retrospective analysis of the study resulted in the identification of several limitations. The calculations of the turmeric concentration were inaccurate, as small amounts of undissolved insoluble filtrate sedimented to the bottom of the solution prior to pipetting. This would decrease the perceived accuracy of the turmeric concentration during the pipetting step. Additional steps of filtration could be carried out to improve the accuracy. 

Another limitation affecting the accuracy and applicability of the results reached was the small sample size which consisted of only five participants from the 4th year dentistry cohort. Small sample size has the potential to increase the margin of error and reduce the confidence interval of the results, reducing the ability to draw definitive conclusions [20]. The sample population of this study (4th year dental students) is not only limited in number but is likely to have a higher standard of oral hygiene practices, due to background knowledge in dentistry. Therefore, plaque samples may not be a true representation of the general population. Additionally, the sample size was limited in diversity of age, race, and gender, which makes it difficult for this study to draw any major conclusions. Furthermore, each individual’s oral cavity has a diverse range and species of bacteria. These bacteria may respond differently to the effects of chlorhexidine gluconate 2 mg/mL which is mainly effective on supragingival plaque, rather than subgingival plaque [21]. Whilst there are genetic variations of each individual’s oral bacteria, this study was not able to identify specific strains of bacteria present in each sample due to lack of sophisticated technology. The operators of this research also had limited background experience in statistics and microbiology which may potentially lead to errors in methodology, data collection, and analysis. This includes stages of the experiment such as pipetting, transferring data onto electronic programs, and mathematically analyzing the results. As such, the results of this study are preliminary and need further corroboration before the findings can be applied.

The results of this study can be implemented as a tool for education purposes for those who use CAMs on a regular basis. Although curcumin itself has been shown to be an effective antimicrobial and anti-inflammatory agent, turmeric powder as a whole has inferior antibacterial properties than pure curcumin on its own. As such, homemade solutions of turmeric mouth rinses are unlikely to have significant antibacterial effects. 

## 6. Conclusions

This in-vitro study evaluated the effects of varying concentrations of turmeric on the growth of oral bacteria. The results demonstrated that turmeric exhibited poor antibacterial properties, with a higher rate of bacterial growth compared to that observed from bacteria in the growth medium alone. Hence, homemade solutions of turmeric mouthwashes are unlikely to have significant antibacterial effects. However, due to limited sample size, it is difficult to extrapolate these results onto a larger scale population, and therefore, further randomized controlled trial studies are required to strengthen the findings of this research.

## Figures and Tables

**Table 1:** Criteria for volunteers for plaque collection.

Inclusion Criteria	Exclusion Criteria
(1) Healthy, consenting James Cook University dental students	(1) Individuals who do not consent (2) Individuals below the age of 18 (3) Individuals who have used broad spectrum antibiotics within the last 3 months. (4) Individuals who have used an antimicrobial mouth rinse within the last two weeks. (5) Individuals who have a known systemic condition (6) Pregnant women

**Table 2:** Layout of the microtiter plate for absorbance analysis. 100 µL was removed from each culture and added to wells in the same organization as above, with the exception of the last row which contained the treatments alone in LB. Duplicate samples were taken from each culture.

Participant		1	2	3	4	5	6	7	8	9	10
1	A	LB	LB	CS	CS	TS-1	TS-1	TS-0.5	TS-0.5	TS-2	TS-2
2	B	LB	LB	CS	CS	TS-1	TS-1	TS-0.5	TS-0.5	TS-2	TS-2
3	C	LB	LB	CS	CS	TS-1	TS-1	TS-0.5	TS-0.5	TS-2	TS-2
4	D	LB	LB	CS	CS	TS-1	TS-1	TS-0.5	TS-0.5	TS-2	TS-2
5	E	LB	LB	CS	CS	TS-1	TS-1	TS-0.5	TS-0.5	TS-2	TS-2
No Bacteria		LB	LB	CS	CS	TS-1	TS-1	TS-0.5	TS-0.5	TS-2	TS-2

LB: Luria-Bertani; CS: Chlorhexidine gluconate 2 mg/mL Savacol; TS: Turmeric Solution.

**Table 3:** Table displaying the calculated exponential growth rates of the oral bacteria for each treatment solution, in OD_600_/hour.

Solution or Treatment	EGR for First Round (in OD_600_/Hour) with Standard Deviation	EGR for Repeat (in OD_600_/Hour) with Standard Deviation
Growth medium alone	0.2443678 +/− 0.14458229	0.2462796 +/− 0.1252849
Chlorhexidine gluconate 2 mg/mL Savacol	0.06366875 +/− 0.04318335	0.0544868 +/− 0.04018672
Turmeric 1× conc.	0.4188425 +/− 0.06607995	0.4702462 +/− 0.12840024
Turmeric 0.5× conc.	0.35589975 +/− 0.19216511	0.4276016 +/− 0.23108732
Turmeric 2× conc.	0.61372325 +/− 0.40408808	0.6232156 +/− 0.35059365

## Data Availability

Restrictions apply to the availability of these data. Data was obtained from dental student and are available from the authors with the permission of Human Ethics committee at James Cook University.
